# Mode-Locked Fiber Laser Sensors with Orthogonally Polarized Pulses Circulating in the Cavity

**DOI:** 10.3390/s23052531

**Published:** 2023-02-24

**Authors:** Hanieh Afkhamiardakani, Jean-Claude Diels

**Affiliations:** 1 Thorlabs, Inc., 56 Sparta Ave., Newton, NJ 07860, USA; 2 Center for High Technology Materials, University of New Mexico, Albuquerque, NM 87106, USA; 3 Department of Physics and Astronomy, University of New Mexico, Albuquerque, NM 87106, USA

**Keywords:** intracavity phase interferometry, laser sensors, sensitivity enhancement, precision sensing, inertial sensors, gyroscopes, ultrafast

## Abstract

Intracavity phase interferometry is a powerful phase sensing technique using two correlated, counter-propagating frequency combs (pulse trains) in mode-locked lasers. Generating dual frequency combs of the same repetition rate in fiber lasers is a new field with hitherto unanticipated challenges. The large intensity in the fiber core, coupled with the nonlinear index of glass, result in a cumulative nonlinear index on axis that dwarfs the signal to be measured. The large saturable gain changes in an unpredictable way the repetition rate of the laser impeding the creation of frequency combs with identical repetition rate. The huge amount of phase coupling between pulses crossing at the saturable absorber eliminates the small signal response (deadband). Although there have been prior observation of gyroscopic response in mode-locked ring lasers, to our knowledge this is the first time that orthogonally polarized pulses were used to successfully eliminate the deadband and obtain a beat note.

## 1. Introduction

### 1.1. Mode-Locking and Frequency Combs

Mode-locking is a popular method for generating frequency combs in which a short pulse is shaped at each round trip of a laser as a result of the balance between group velocity dispersion and self-phase modulation (SPM) in the laser cavity. The circulating intracavity pulse creates, through an output coupler, a train of pulses equally spaced in time, whose Fourier transform provides equally spaced teeth, which constitute a comb in the frequency domain. Optical frequency combs have dramatically improved the accuracy of frequency metrology [1]. Various applications of frequency combs such as atomic clock [2], calibration of astronomical spectrographs [3], comb-based spectroscopy [4], distance measurement, and laser ranging are based on absolute frequency measurement. In all these applications, relying on *single* frequency combs, the absolute value of the carrier to envelope offset (CEO) frequency, a very important parameter in comb characterization, is required to achieve high-precision frequency metrology. The CEO of a single frequency comb is only a meaningful quantity if the frequency comb is stabilized (In an unstabilized laser, the random fluctuations of the CEO frequency may exceed the mode spacing, making it totally undefined).

Stabilization adds significantly to the price of a mode-locked laser. The optical frequency of a tooth of a stabilized frequency comb locked to frequency standards can be determined with an accuracy of 1 mHz [5]. With 1 mHz tooth bandwidth, these systems can detect a change in optical frequency with a resolution of $1:10^{18}$. Such stabilization is overkill for a sensor based on differential measurements between two unstabilized frequency combs issued from the same laser. We refer to this differential comb interferometry as intracavity phase interferometry (IPI). Despite the broad bandwidth of each individual comb, beat note bandwidth of less than 0.01 Hz bandwidth has been reported [6]. This phase measurement corresponds to a phase uncertainty of 0.4 $\times \;10^{-9}$ radian, or a phase-photon number uncertainty product of 0.66, close to the quantum limit of 0.5. Another recent results pertaining to IPI is the possibility of passive stabilization with a low finesse resonator (Fabry Perot) inserted in the mode-locked laser [7]. It has also been shown that the beat note frequency can be increased by coupling the cavity to a Gires–Tournois [8] or a ring resonator [9]. These results were obtained with Ti:sapphire lasers and Ti:sapphire lasers pumped optical parametric oscillators (OPO), which are cumbersome, large, and inefficient systems. Our goal is to achieve similar performances with compact and low power consumption fiber based systems.

### 1.2. Mode-Locked Lasers for IPI

The main concern in IPI design is generating two uncoupled pulse trains with the same repetition rate in a laser cavity. Two methods are possible: (i) gating the gain, as in optical parametric oscillators (OPO), or (ii) gating through the losses, as with saturable absorbers. The former method has been mainly used in discrete components lasers [10]. The use of saturable absorbers has been limited by a large deadband [11]. We have devised a method to eliminate any phase coupling between the circulating pulses by circulating orthogonally polarized pulses in the ring cavity.

Whether to use a ring or linear cavity for IPI depends on the quantity to be measured. The principle of the measurement is the same in both laser designs. In IPI, the physical quantity to be measured (flow velocity, electric field, magnetic field, rotation, acceleration, and displacement) imparts a phase shift Δϕ between the two intracavity pulses per round trip, which results in a frequency shift of [12]:

(1) $$\Delta \nu =\frac{\Delta \phi }{2\pi \tau_{rt}}=\nu \frac{\Delta P}{P},$$

where $\tau_{rt}$ is the cavity round-trip time at the phases velocity, ν is the frequency of light, *P* is the optical perimeter of the ring laser cavity (twice the cavity length in the case of a linear cavity), and ΔP is the difference in optical path length that would correspond to the phase difference Δϕ between two frequency combs. The frequency change of Δν is measured as a beat note by interfering two pulse trains (frequency combs) outside the laser cavity.

The bandwidth of the beat note can be as small as 0.01 Hz [6], even though each tooth comb has a bandwidth larger than 1 MHz, again indicating the correlation between combs. This amount of bandwidth corresponds to a phase shift of $\approx 0.4\times 10^{-9}$ rad or optical path length change of 0.07 fm. The sensitivity of the intracavity measurement is thus typically seven to eight orders of magnitude better than the resolution of a Michelson interferometer.

### 1.3. IPI in Fiber Lasers

Implementing IPI in fiber lasers is closer to commercial products in terms of simplicity and cost. Creating a fiber laser with two intracavity pulses circulating at the same group velocity is a challenge addressed in this paper. The main hurdle resides in the strong intensity dependence of group velocity inside a fiber laser [13], which leads to unequal repetition rate for the two circulating pulses. The solution for forcing the two pulses to circulate at the same cavity round-trip time is found in the design of a symmetric cavity, a solution that also minimizes the “bias beat note” (spurious response). It is shown (experimentally and theoretically) that the pulse velocity in the cavity is dominated by gain dynamics, and it is not simply equal to the group velocity [13,14].

A practical application of IPI is in inertial navigation systems comprising accelerometers (linear IPI) and laser gyros where the phase shift to be measured is the Sagnac phase shift. Because short pulses are circulating in the cavity, they can only scatter into each others at their crossing point. Therefore, a mode-locked gyroscope does not generally have a deadband [12], as its classical cw He-Ne counterpart. However, when the pulse crossing control is implemented with a non-moving saturable absorber, the scattering at that point causes injection locking of each beam into the other. As a result, there is no response at low rotation rates (deadband region). Polarization maintaining (PM) fibers offer a solution to that problem, because the counter-circulating pulses can be prepared with orthogonal polarization, being linearly polarized along the slow and fast axes. The coupling at the crossing point is therefore minimized. An original method to create a ring laser where the counter-circulating pulses are cross-polarized is devised and described here. Its successful implementation paves the way for the future development an all-fiber active laser gyro.

### 1.4. Passive Mode-Locking in Fiber Lasers

In passive mode-locking, a nonlinear passive element (saturable absorber) causes the light in the laser resonator to be amplitude modulated, leading to the formation of an ultrashort pulse. Ultrafast erbium-doped fiber lasers can be passively mode-locked using a variety of nonlinear materials and components, such as semiconductor saturable absorber mirrors (SESAMs) [15], carbon nanotubes (CNTs) [16], graphene [17], topological insulators [18], and nonlinear optical/amplifying loop mirrors (NOLM/NALM) [19]. Multi-wall and single-wall carbon nanotubes (CNTs) were first discovered by Sumio Iijima in 1991 [20] and 1993 [21], respectively. It was soon realized that single-wall CNTs can be either metallic or semiconducting depending on their structure (chirality) [22], and possess very fast (<1 ps) saturable absorption [23] being applied in mode-locking of fiber lasers [16]. CNT has therefore become a very popular nonlinear material for mode-locking of fiber lasers because of several advantages such as the possibility of making simple, compact, in-line, and fast saturable absorbers. Numerous reports have been published on unidirectional mode-locked fiber lasers using single mode fibers incorporating carbon nanotubes [24,25,26,27,28,29]. As the polarization of light propagating in a single mode fiber is very sensitive to external perturbations, there is a desire to apply this mode-locking technique to polarization maintaining (PM) fiber lasers. There are only a few reports on all-PM erbium-doped fiber lasers mode-locked by CNTs. Nishizawa et al. demonstrated an all-PM Er-doped ultrashort-pulse fiber laser using a CNT-polyimide film as a saturable absorber [30]. An all-PM fiber ring laser reported by Jeong et al. [31] uses a side-polished (D-shaped) PM fiber coated with CNT/polymer composite.

**Figure 1:**
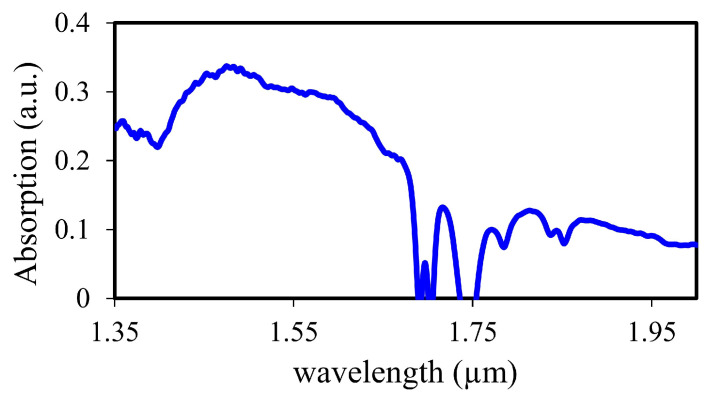
Absorption spectrum of carbon nanotubes.

The characterized carbon nanotubes used in this work have tube diameters from 0.9 nm to 1.5 nm, and tube lengths from 0.3 μm to 4 μm. In Figure 1, the absorption spectrum of CNTs shows a significant absorption of light at 1.55 μm, which is usually the operating wavelength of erbium-doped fiber lasers. Our CNT-based saturable absorber were manufactured by inserting CNTs between two fiber ferrules [26].

### 1.5. Deadband in IPI

A mode-locked laser for IPI has to generate two pulses with the same repetition rate. This condition comes true only if pulses meet at the same location in the cavity at each round trip. The mutual saturation of the saturable absorber (SA) forces two pulses propagating in the cavity to cross at a predetermined location of the SA [12]. This causes phase coupling with the back-scattered light from the SA to create a deadband region in the plot of beat note response versus phase difference or mode splitting.

One possible solution to avoid phase coupling between pulses at the saturable absorber is to generate orthogonally polarized pulses in the laser cavity. Mode-locked polarization maintaining (PM) fiber lasers are the most promising lasers towards deadband-free IPI, because the two pulses can be made to propagate along the slow axis and the fast axis: two orthogonally polarized, non interacting, cavities.

## 2. Implementation of IPI in Mode-Locked Ring Fiber Lasers

### 2.1. Parallel Polarization

Two main applications of IPI in ring configuration are magnetometry [32] and rotation sensing. In the latter application, the differential phase created by a rotation angular frequency $\Omega_{r}$ is the Sagnac [33,34] phase shift $\Delta \phi =8\pi A\Omega_{r}/\left(\lambda c\right)$, which, when inserted in Equation (1), leads to a beat note response:

(2) $$\Delta \nu =\frac{4A}{P\lambda }\Omega_{r},$$

where λ and *c* are the wavelength and speed of light, and *A* is the surface enclosed by the perimeter *P*. In contrast to all other IPI applications where miniaturization is desirable because the response Δν is *inversely proportional* to the linear dimensions, Equation (2) shows the response to be *proportional* to the linear dimensions. Fiber lasers are therefore particularly attractive for this application, because a large perimeter can be achieved with minimal weight and volume. Colliding pulse mode-locking in a bidirectional discrete component ring laser creates two counter-propagating pulses that meet at the same location established by the saturable absorber at each round trip [35,36,37,38]. This is generally not the case in ring fiber lasers, where gain saturation results in different group velocities for the counter-circulating pulses [13], an effect that dwarfs the colliding pulse effect. The solution to this problem is to take extreme care to design a rigorously symmetric structure of a bidirectional fiber laser, as was done in [13]. Rather than using tapered fiber saturable absorbers, better localization of the pulse crossing point is achieved with the carbon nanotubes sandwiched between two FC/APC fiber connectors [26]. A similar symmetric structure was used as in [13] with two portions of Er-doped fibers from Nufern (PM-ESF-7/125) disposed on either side of the saturable absorber. The cavity can be made perfectly symmetric by placing the 2 × 2 output coupler (OC) at the crossing point of the two pulses opposite to that of the saturable absorber. That introduces another source of coupling between the two pulses, as they cross at that location. To eliminate this coupling (both at the saturable absorber and at the opposite crossing point), the two counter-circulating pulses are made orthogonally polarized, as described in the next section.

### 2.2. Orthogonal Polarization

Coupling by backscattering has to be prevented or minimized in the saturable absorber where the pulses cross. In a fiber laser, the amount of coupling between pulses at the crossing point can be reduced by making pulses cross-polarized.

**Figure 2:**
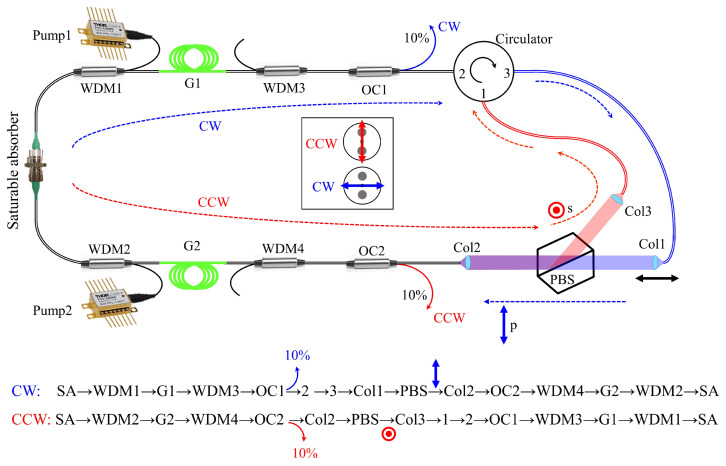
Schematic of the PM fiber laser cavity generating two cross-polarized counter-circulating pulses. WDM: wavelength division multiplexer, G: gain fiber, OC: output coupler, Col: collimator, PBS: polarizing beam splitter, s: s-polarized, p: p-polarized.

The cavity configuration having two circulating pulses orthogonally polarized is shown in Figure 2. The all-PM fiber laser has two portions of Er-doped gain fibers (G1 and G2) pumped through WDM1 and WDM2 by laser diodes at 980 nm, labeled as Pump1 and Pump2 in Figure 2. The other two WDMs (WDM3 and WDM4) are used to trap the radiation of pumps in the region of gain fibers to isolate the rest of the laser from 980 nm radiation. The saturable absorber is based on the interaction of light with a thin layer of CNTs between two fiber ferrules, resulting in colliding pulse mode-locking. A combination of a 3-port circulator and a lossless polarizing beam splitter (PBS) forces unidirectional operation of s-polarized light in the CCW direction and p-polarized light in the CW direction. In the CCW direction, the light is polarized along the slow axis of the PM fiber, whereas in the CW direction, the light is polarized along the fast axis, as shown in the inset of the Figure 2.

The PBS is designed and made to reflect the s-component of the light in the CCW direction and to transmit the p-component in the CW direction. The pair of collimators (Col1 and Col2 in the CW direction and Col2 and Col3 in the CCW direction) are responsible for making collimated beams in air for transmission/reflection at the PBS. The alignment of the free-space part is extremely critical to achieve bidirectional mode-locking. The collimators did not provide a perfectly collimated beam, but a slightly converging one with a waist at ≈15 cm. Therefore, not only are their orientation and transverse positioning critical, but also their longitudinal spacing. Each of the three collimators is mounted on a separate 6-axis positioner including rotation to adjust the orientation of PM fibers at the three collimators. An overall transmission factor of 95% through the PBS was achieved. The 3-port circulator (1→2→3) guarantees the unidirectional operation of cross-polarized beams. The s-polarized light is guided through port 1 to port 2 of the circulator, and p-polarized light in the CW direction is transmitted from port 2 to port 3 of the circulator.

**Figure 3:**
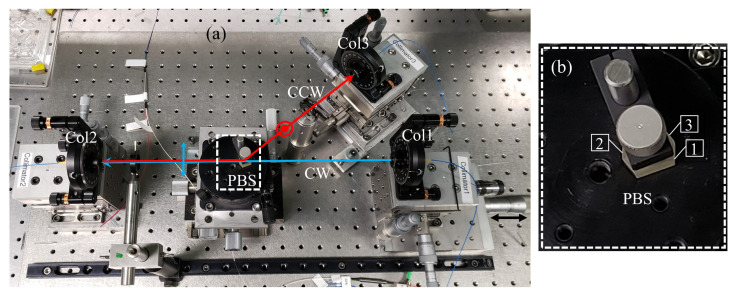
(**a**) The experimental setup of the free-space part of the orthogonally polarized ring fiber laser to separate the two orthogonal polarizations. Col: collimator, PBS: polarizing beam splitter. (**b**) The magnified picture of the PBS with the three optical faces labeled.

The cavity length in either direction is controlled by translating Col1 to ensure that the optical cavity length is equal in both directions. The path of light in either direction, starting from the saturable absorber, is explained at the bottom of Figure 2. Figure 3a shows the actual setup of the free-space part of the laser in Figure 2. The paths of light in CW and CCW directions are sketched in blue and red, respectively.

The 10% output couplers (OC1 and OC2) are placed symmetrically with respect to the saturable absorber to extract 10% of light in either direction. Therefore, the gain and losses at both sides of the absorber can be equal (in the case of pumping the same lengths of gain fibers equally) and result in comparable output powers on each direction.

The magnified picture of the PBS is shown in Figure 3b with the faces labeled. It is made of two YVO_4_ crystals (positive uniaxial) optically contacted. The one with face (1) in Figure 3b has its optics axis parallel to the incident beam, whereas the other one has its axis orthogonal to the plane of the figure. As a result, the p-component of the beam entered from surface 1 is an ordinary ray for both prisms, and is transmitted without loss. The s-component of the beam incident to surface 2 undergoes total internal reflection at the interface, being extraordinary ray (higher index) in the upper prism, and thus does not suffer any loss. The loss in p-transmission and s-reflection were both measured to be less than 1%.

### 2.3. Beat-Note Measurement

The two outputs of the laser in the CW and CCW directions are orthogonally polarized as expected. In order to measure the beat note, the two directions of circulation should have exactly equal round trip time. Because the indices of refraction along the fast (CW direction) and slow axes (CCW direction) of the PM fiber are different, the optical length is not measured as purely geometrical length. The position adjustment of the collimators (Col1, Col2, and Col3 in Figure 2) is therefore very critical to have the same repetition rate in each direction.

**Figure 4:**
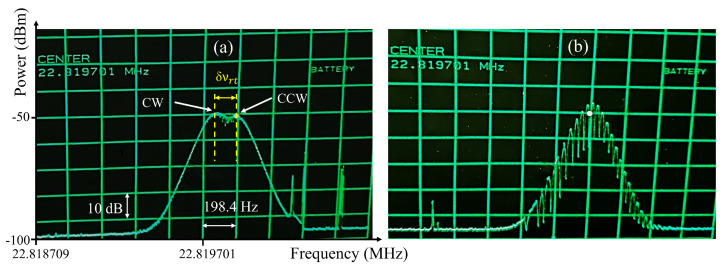
Radio frequency spectrum of the pulse trains in opposite directions of the cross-polarized fiber laser with (**a**) different and (**b**) the same repetition rates. The scales on the x and y axes are the same in both graphs. CW: clockwise, CCW: counter-clockwise, bandwidth resolution: 100 Hz.

First, all collimators should be set for the best coupling ratio in each direction, then by translating Col1 (while maintaining alignment), the equal repetition rate condition may be found. Figure 4a shows the radio frequency (RF) spectra of pulse trains in the CW and CCW directions, when the repetition rates are not equal, and pulses in the CW direction have a lower repetition rate. In this figure, $\delta \nu_{rt}$ shows the difference between repetition rates. To match the rates, Col1 in Figure 2 has to be moved to the left to shorten the cavity length and increase the repetition rate of pulses in the CW direction by $\delta \nu_{rt}$. This method helps to learn in which direction the collimator (Col1) should be moved. Once the equal repetition rates are achieved, the RF spectrum changes to the modulated pattern shown in Figure 4b. The latter figure could be understood as a kind of RF spectral interferometry. The optical field on the detector is:

(3) $$E=E_{1}+E_{2}=\sum_{p}E_{1}e^{i(\omega +p\nu_{rt,1})t}+\sum_{q}E_{1}e^{i(\omega +q\nu_{rt,2})t},$$

where ω is the optical frequency, $\nu_{rt,1}$ the mode spacing of comb 1, and $\nu_{rt,2}=\nu_{rt,1}+\epsilon$ the mode spacing of comb 2. The detector records a signal proportional to $|E^{2}|$. The RF spectrum analyzer zooming in the region close to $\nu_{rt,1}$ records the signal $|E_{1}E_{2}^{*}|$ with frequencies at $\nu_{rt,1}+\left(q-p\right)\epsilon$. In the case of Figure 4b, $\nu_{rt,1}=22.820$ MHz and ϵ≈0.000030 MHz. This indicates that we are within 10 μm of equal perimeter for both directions.

**Figure 5:**
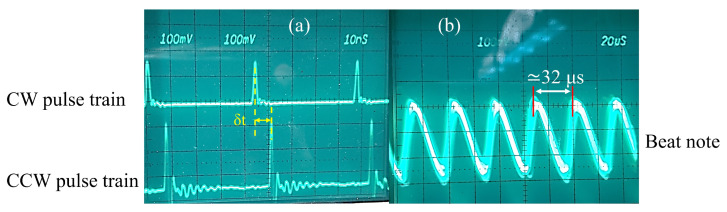
(**a**) Oscilloscope trace of the clockwise (upper) and counter-clockwise (lower) pulse trains in cross-polarized fiber laser. (**b**) The envelope of the modulated pulse train due to the asymmetry in the ring cavity.

An external delay line is manufactured to compensate for the time delay δt (Figure 5a) between output pulses in opposite directions in order to measure the beat note frequency. A RF low pass filter has been used to filter the high frequency pulses keeping the low frequency envelope of the modulated pulse train, as illustrated in Figure 5b. By taking the inverse of the modulation period (≈32 μs) in this figure, the beat note frequency is calculated as ≈31 kHz. With a cavity perimeter of $c/n\nu_{rt}$ ($\nu_{rt}=22.82$ MHz and n=1.44) or ≈9 m, we find from Equation (1) that the difference in cavity length for the two polarizations is only 1.5 nm! The bulk arrangement of Figure 3 is not amenable for reproducibly achieving nanoaccuracy. Our results, however, indicate that the concept is valid, and that it is worth investing in an integrated optics version of the arrangement of Figure 3. Obviously, considerably more elaborate mechanical isolation/rigidity is needed to exploit the sensitivity of this method. This results is most likely also applicable to linear IPI, where the geometry is more amenable to a rigid interferometer.

This is an initial measurement without optimizing pump powers to lower the amount of measured beat frequency. However, a beat note of 31 kHz is measurable in cross-polarized ring fiber laser, which is almost 32× less than the lowest measurable beat frequency (≈1 MHz) in parallel polarization. It means that cross-polarized pulses in the ring fiber laser have lowered the phase coupling significantly at the crossing point (saturable absorber).

## 3. Conclusions

Fiber lasers seem to be ideal devices for intracavity phase interferometry (IPI), because the beam confinement protects it from external influences with particular application in rotation sensing as an active laser gyroscope. However, IPI in fiber lasers has faced unexpected challenges. The first one is the difficulty in imposing the same average group velocity for the two pulses circulating in the laser cavity. This causes different repetition rates for counterpropagating pulses, which can be resolved by designing a symmetric cavity in which two gain sections are located symmetrically with respect to a single carbon nanotube saturable absorber responsible for mode-locking. The bias beat note can be fine-tuned by adjusting the pump powers in the two gain sections. Another challenge specific to passively mode-locked fiber lasers is the coupling between the two intracavity pulses at the saturable absorber, which leads to a large deadband (absence of response at low beat note frequencies). A new method of eliminating the coupling between counter-circulating pulses in a fiber laser gyro is devised, by which the two intracavity circulating pulses are given orthogonal polarization. The two pulses are giving separate paths by a combination of circulator and polarizing beam splitter. The extreme stability and precision that is required to make the two path rigorously equal can only be achieved by creating an integrated optics version of the polarization split arrangement shown in Figure 3. Such nanotechnology was not available and is beyond the scope of this article, which aimed to demonstrate the viability of the polarization splitting in a fiber laser gyro. A bias beat note of only 31 kHz was recorded, implying that we were able to adjust the cavity lengths in both directions to be equal within 2 nm. This is a remarkable result, given that the free-space part of the laser pictured in Figure 3 is extremely sensitive to the environmental conditions such as vibration, temperature, air current, etc… These preliminary measurements constitute a proof of principle of the validity of a fiber laser gyroscope mode-locked by carbon nanotube saturable absorber.

## Data Availability

The data presented in this study are available on request from the corresponding author.

## Abbreviations

The following abbreviations are used in this manuscript:

CW	Clockwise
CCW	Counterclockwise
PM	Polarization Maintaining
CNT	Carbon Nanotube
PBS	Polarizing Beam Splitter
OC	Optical Coupler
WDM	Wavelength Division Multiplexer
SA	Saturable Absorber
Col	Collimator
G	Gain
RF	Radio Frequency
IPI	Intracavity Phase Interferometry
Gyro	Gyroscope
CEO	Carrier Envelope Offset
